# Proteomic signatures of intermittent pneumatic compression in patients with large artery atherosclerotic stroke

**DOI:** 10.1016/j.isci.2026.114915

**Published:** 2026-02-10

**Authors:** Shasha Lei, Zhenzhen Wang, Dandang Ouyang, Tingjing Huang, Kaili Huang, Xinying Li, Zhi-Xin Huang

**Affiliations:** 1 Department of Neurology, The Affiliated Guangdong Second Provincial General Hospital of Jinan University, Guangzhou 510317, China; 2 Department of Neurology, The Fifth Affiliated Hospital of Southern Medical University, Guangzhou, Guangdong 510900, China; 3 Guangdong Business and Technology University, Zhaoqing 526020, China; 4 LC-Bio Technologies, Co., Ltd, Hangzhou 310018, China

**Keywords:** health sciences, medicine, medical specialty, cardiovascular medicine

## Abstract

Intermittent pneumatic compression (IPC) is routinely used after stroke for venous thromboembolism prophylaxis, yet its molecular mechanisms remain elusive. In a single-blind randomized pilot trial, 44 patients with large-artery atherosclerotic ischemic stroke were assigned to bilateral IPC twice daily (*n* = 22) or unilateral compression once daily (*n* = 22) for 7 (SD = 2) days. Cardiovascular proteins were quantified with the Olink platform, and treatment-specific changes were identified using a prespecified triple orthogonal screening strategy. IPC was associated with 12 treatment-specific proteins, 93.3% of which increased after intervention. ANGPT1 and SOD2 were prominent candidates, mapping to angiogenesis, antioxidant, and inflammation pathways, including Ras signaling, correlating with NIHSS and ESRS scores. These hypothesis-generating data suggest IPC modulates defined protein networks (Ras-ANGPT1-related and SOD2-mediated antioxidant pathways) that may support mechanistic and precision rehabilitation studies, pending validation in larger cohorts.

## Highlights

• Ischemic preconditioning (IPC) alters levels of 12 circulating proteins in patients with stroke
• IPC-induced protein changes correlate with neurological outcomes and vascular risk scores
• IPC modulates proteins in oxidative stress, inflammation, and vascular remodeling pathways
• Angiopoietin-1 (angiogenic) and SOD2 (antioxidant) emerge as key proteins upregulated by IPC

## Graphical abstract

**Figure:**
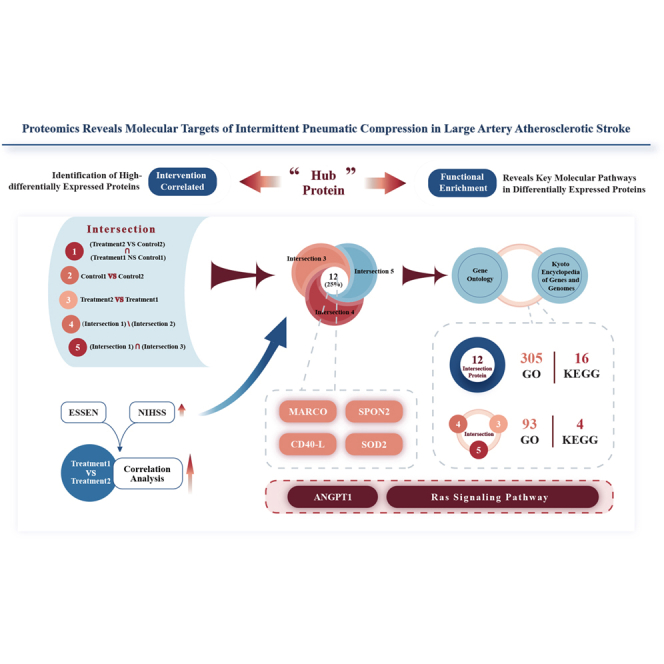

## Introduction

Stroke represents a formidable global health challenge, ranking as the third leading cause of death and the fourth leading cause of disability worldwide, affecting an estimated 93.8 million individuals in 2021.^1^ Large artery atherosclerotic (LAA) stroke—the predominant ischemic stroke subtype comprising 30–40% of cases—is characterized by significant stenosis or occlusion of major cerebral arteries and associated with more severe neurological deficits and elevated recurrence risk.^2^ Despite revolutionary advances in acute management, approximately 50–70% of survivors experience persistent neurological deficits that profoundly impact the quality of life and strain healthcare systems.^3^ Current rehabilitation paradigms rely predominantly on physical, occupational, and speech therapies, with limited biological interventions targeting underlying pathophysiological mechanisms. Many patients demonstrate suboptimal recovery, highlighting an urgent need for innovative therapeutic strategies that enhance neurological restoration through multi-mechanistic approaches.^4^^,^^5^^,^^6^

Intermittent pneumatic compression (IPC), traditionally utilized for venous thromboembolism prophylaxis, demonstrates potential benefits extending beyond thromboprophylaxis, possibly influencing cerebral hemodynamics and neurological recovery in patients with stroke.^7^ This neuroprotective effect is thought to arise from a mechanism wherein IPC—a device that applies intermittent pressure to limbs via cyclical inflation and deflation of air cuffs^8^—induces intermittent micro-ischemia in the limbs. This process, in turn, triggers ischemic preconditioning, putting the brain into a tolerant state and enhancing its resistance to subsequent severe ischemic insults, thereby helping mitigate post-stroke ischemic brain damage.^9^^,^^10^ However, the molecular mechanisms underlying these effects remain largely unexplored, creating a critical knowledge gap in understanding how mechanical interventions modulate biological processes relevant to stroke recovery. This gap is particularly noteworthy given the emerging recognition of intricate relationships between physical interventions and molecular signaling cascades.^11^^,^^12^^,^^13^ While pharmacological effects on stroke-related protein expression have been extensively characterized,^14^^,^^15^^,^^16^ limited knowledge exists regarding how rhythmic compression influences the proteomic landscape in patients with stroke.

Existing IPC research, particularly in non-stroke populations, has predominantly examined macro-hemodynamic responses rather than downstream molecular correlates. Clinical and experimental studies in athletes, surgical patients, and animal models consistently show that IPC protocols acutely alter limb blood-flow velocity, venous return, and systemic circulatory parameters, supporting its role in optimizing peripheral hemodynamics and reducing venous stasis.^17^^,^^18^^,^^19^ However, IPC-related proteomic changes in stroke populations—especially in patients with large artery atherosclerotic stroke—have been scarcely described. Our study, therefore, provides an initial, hypothesis-generating proteomic characterization of IPC in this population, aiming to identify IPC-associated protein signatures that may inform future mechanistic investigations.

Our investigation explores IPC’s effects on cardiovascular-related protein expression in patients with LAA stroke using comprehensive proteomics. We aimed to determine: (1) whether IPC induces significant alterations in cardiovascular-related protein expression; (2) which specific proteins and pathways are modulated; and (3) whether these molecular changes correlate with clinical outcomes. The significance of this research lies in its potential to elucidate IPC’s biological foundation in stroke management, potentially transforming our understanding from a purely mechanical therapy to a biologically active treatment with specific molecular targets.

## Results

### Participant characteristics

Table 1 summarizes the demographic and clinical baseline characteristics of study participants. The treatment group (*n* = 22) and the control group (*n* = 22) were well-balanced at baseline. Treatment group participants averaged 62 (SD = 12) years, comprising 11 males (50.0%) and 11 females (50.0%). Control group participants averaged 60 (SD = 13) years, with 18 males (81.8%) and 4 females (18.2%). No significant between-group differences emerged regarding age (*p* = 0.601), sex distribution (*p* = 0.056), body mass index (BMI) (*p* = 0.899), or other baseline clinical characteristics (infarct volume, infarct location, National Institutes of Health Stroke Scale (NIHSS) score at admission, systolic and diastolic blood pressure at admission, Alberta Stroke Program Early CT Score (ASPECTS)). Importantly, no intervention-related adverse events occurred throughout the study period, confirming IPC’s safety profile. Further analysis showed that protein expression levels were not significantly correlated with sex in our study cohort. Moreover, the core proteins identified through our screening process did not overlap with proteins that exhibit sex-related differences in expression. Additionally, analyses of post-treatment samples confirmed that sex factors did not influence the expression patterns of the key proteins identified in this study. Thus, sex is not a major confounding factor in our findings. Detailed results are provided in the Tables S1 and S2.

**Table 1:** Baseline characteristics of study participants

Characteristic	Control (*n* = 22)	Treatment (*n* = 22)	*p*-value
**Demographic characteristics**			

Age, years	60.18 (12.63)	62.14 (11.95)	0.601
Female sex, n (%)	4 (18.2)	11 (50.0)	0.056
BMI, kg/m^2^	24.00 (4.03)	23.86 (3.50)	0.899

**Lifestyle factors**			

Smoking, n (%)	10 (45.5)	7 (31.8)	0.536
Alcohol consumption, n (%)	4 (18.2)	3 (13.6)	1.000

**Clinical parameters**			

Systolic BP on admission (mmHg)	145.50 (24.39)	157.64 (26.02)	0.118
Diastolic BP on admission (mmHg)	83.23 (12.21)	84.55 (13.23)	0.733
NIHSS score	5 (4)	7 (3)	0.062
ASPECTS score	3.41 (1.44)	4.05 (1.21)	0.119
DWI infarct volume, cm^3^	18.12 (11.73)	21.95 (9.49)	0.241

**Medical history, n (%)**			

Hypertension	18 (81.8)	18 (81.8)	1.000
Diabetes mellitus	9 (40.9)	10 (45.5)	1.000
Previous stroke	9 (40.9)	5 (22.7)	0.332
Coronary heart disease	3 (13.6)	1 (4.5)	0.600
Hyperlipidemia	1 (4.5)	1 (4.5)	1.000

Data are presented as mean (SD) or n (%) unless otherwise specified. *p*-values were calculated using Student’s *t* test for continuous variables and Fisher’s exact test or chi-squared test for categorical variables.

BP: blood pressure; BMI: body mass index; NIHSS: National Institutes of Health Stroke Scale; ASPECTS: Alberta Stroke Program Early CT Score; DWI: diffusion-weighted imaging.

### Comprehensive proteomic profiling reveals treatment-specific signatures

**Figure 1:**
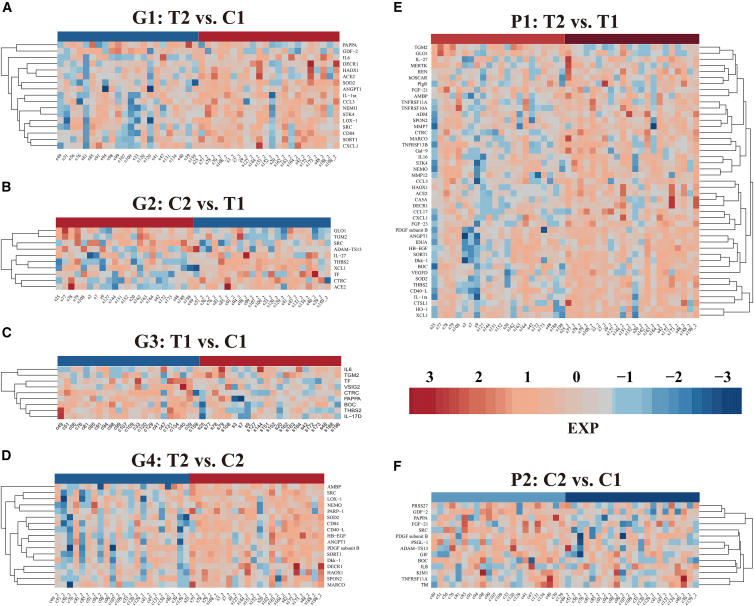
Heatmap analysis of differentially expressed cardiovascular-related proteins across multiple comparison groups (A) Comparison between post-treatment and control baseline groups (T2 vs. C1), showing 17 DEPs (15 upregulated, 2 downregulated). (B) Differential protein expression between post-control and treatment baseline groups (C2 vs. T1), with 10 proteins showing significant changes (6 upregulated, 4 downregulated). (C) Baseline comparison between treatment and control groups (T1 vs. C1), identifying 9 DEPs (2 upregulated, 7 downregulated). (D) Post-intervention comparison (treat2 vs. ctrl2), revealing 17 DEPs (all upregulated in the treatment group). (E) Paired longitudinal analysis within the treatment group (T2 vs. T1), showing the most extensive changes with 45 significantly altered proteins (42 upregulated, 3 downregulated). (F) Paired analysis within the control group (C2 vs. C1), with 14 DEPs (2 upregulated, 12 downregulated). Red indicates upregulation and blue indicates downregulation. Sample IDs shown at bottom, protein names displayed on right. Abbreviations: DEPs, differentially expressed proteins.

**Figure 2:**
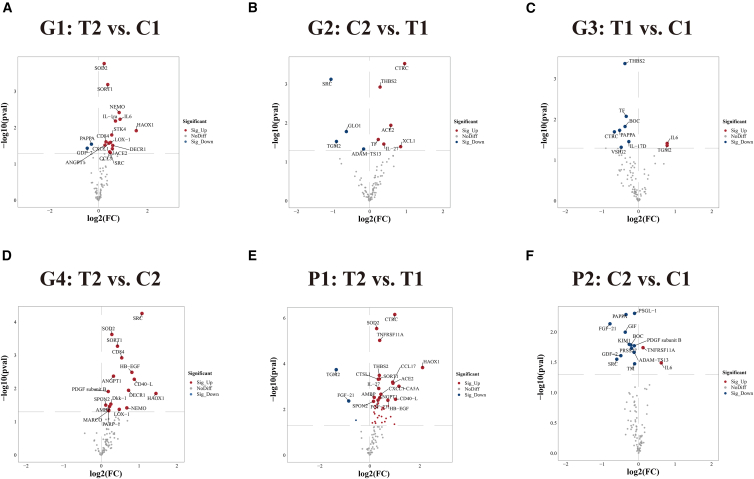
Volcano plots of differentially expressed cardiovascular-related proteins across six comparison groups (A) Treatment post-intervention (T2) vs. control baseline (C1): 17 proteins showed significant differential expression, with most upregulated in the treatment group. (B) Control post-intervention (C2) vs. treatment baseline (T1): 10 proteins were differentially expressed with mixed expression trends. (C) Treatment baseline (T1) vs. control baseline (C1): 9 DEPs, predominantly downregulated in the treatment group. Panel C (G3: T1 vs. C1) illustrates baseline differences between randomized groups and is presented for descriptive purposes; treatment-related inferences are based primarily on longitudinal contrasts (G4, P1, P2). (D) Treatment post-intervention (T2) vs. control post-intervention (C2): all 17 proteins were significantly upregulated. (E) Paired comparison within the treatment group (T2 vs. T1): 45 proteins were differentially expressed, with a strong upregulation trend post-treatment. (F) Paired comparison within the control group (C2 vs. C1): 14 proteins were significantly altered, with most showing downregulation after observation. The x axis represents log2 fold change (log2FC), and the y axis represents -log10(*p*-value). Significant proteins (*p* < 0.05, |log2FC|>0.5) are labeled with gene names. Red and blue dots indicate significantly upregulated and downregulated proteins, respectively, while grey dots represent non-significant changes.

Systematic comparative analysis identified multiple differentially expressed cardiovascular-related proteins across six comparison groups. The post-treatment versus control baseline comparison (G1) revealed 17 significantly differentially expressed proteins (DEPs), with 15 (88.2%) demonstrating upregulation post-treatment (treat2) and only 2 (11.8%) showing downregulation (Figures 1A and 2A). Conversely, the post-control versus treatment baseline comparison (G2) identified 10 DEPs, with 6 (60%) upregulated in the post-control group (ctrl2) and 4 (40%) downregulated expression (Figures 1B and 2B).

The treatment baseline versus control baseline comparison (G3) revealed 9 DEPs, with 7 (77.8%) downregulated in the treatment group baseline (treat1) (Figures 1C and 2C), suggesting potential baseline heterogeneity despite randomization. Most significantly, the post-treatment versus post-control comparison (G4) demonstrated that all 17 DEPs (100%) exhibited upregulation in the post-treatment group (T2) (Figures 1D and 2D), a pattern that is compatible with intervention-related biological effects in this exploratory analysis.

Paired longitudinal analyses revealed more pronounced patterns. Within-treatment group analysis (P1) showed significant changes in 45 proteins, with 42 (93.3%) upregulated post-intervention (T2) and only 3 (6.7%) downregulated (Figures 1E and 2E). In contrast, within-control group analysis (P2) identified 14 DEPs, with 12 (85.7%) downregulated post-observation (C2) and only 2 (14.3%) upregulated (Figures 1F and 2F).

The contrasting patterns between treatment and control groups suggest that IPC not only promotes protein upregulation but may also counteract natural protein degradation processes occurring over time in patients with stroke.

### Multi-dimensional screening strategy identifies treatment-specific biomarkers

**Figure 3:**
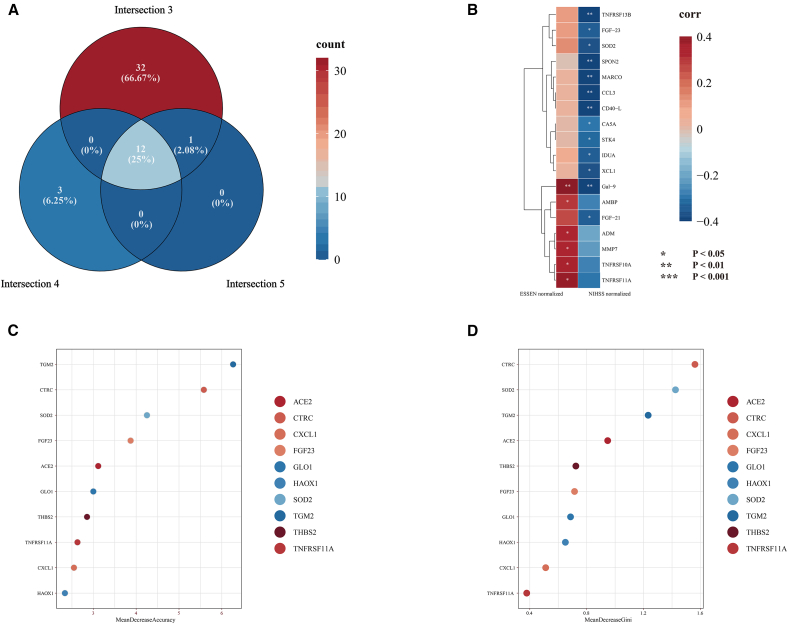
Prioritization and exploratory evaluation of candidate treatment-associated proteins (A) Venn diagram illustrates the overlap between three candidate protein sets derived from the triple orthogonal screening strategy. The final set of 12 treatment-specific core proteins was obtained by overlapping Set 3 (treatment-responsive), Set 4 (excluding time-dependent effects), and Set 5 (shared significance in both intergroup and intragroup comparisons). (B) Heatmap showing Spearman correlation coefficients between vascular-related proteins and clinical scores (NIHSS and ESRS) in the treatment group (P1). Thirteen proteins were significantly associated with NIHSS scores, and six with ESRS scores. ∗ (*p* < 0.05), ∗∗ (*p* < 0.01), and ∗∗∗ (*p* < 0.001). (C) Random Forest analysis ranking the 45 DEPs by mean decrease in accuracy, indicating their relative contribution to the model’s predictive performance. (D) Importance ranking of the same proteins by the mean decrease in Gini index. Notably, SOD2 exhibited top importance in both measures, supporting its central role in the therapeutic response. Color intensity in all heatmaps and plots reflects either frequency (A), correlation coefficient (B), or feature importance (C–D), with red indicating higher values.

This study implemented a triple orthogonal screening strategy to prioritize proteins showing patterns consistent with potential treatment-related modulation (Figure 3A). The approach was designed as a structured, exploratory filter to isolate treatment effects by controlling for baseline heterogeneity and time-dependent changes when selecting candidate proteins for further analysis.

The screening process began with three initial protein sets derived from planned pairwise comparisons. Set 1 included proteins without detectable baseline differences (T1 vs. C1, *p* > 0.05) but with inter-group differences after intervention (T2 vs. C2, *p* < 0.05), highlighting proteins whose post-intervention levels diverged between the IPC and control groups in the absence of obvious baseline imbalance (*n* = 24); Set 2 comprised time-dependent proteins with significant changes in the control group (C2 vs. C1, *p* < 0.05), primarily reflecting the natural course of disease and background temporal variation (*n* = 14); Set 3 included treatment-responsive proteins with significant changes in the treatment group (T2 vs. T1, *p* < 0.05), encompassing direct treatment effects and disease course covariates (*n* = 45).

Further refinement was achieved through intersection analysis. Set 4 was generated by removing proteins from Set 1 that were identified as differentially expressed in Set 2. This step ensured that Set 4 (*n* = 19) specifically captured proteins related to the treatment effect, excluding those that might be attributed to time-dependent disease progression. Set 5 was defined as the intersection of Sets 1 and 3, highlighting proteins that exhibited both inter-group differences at follow-up and longitudinal change in the treatment group (*n* = 16).

Finally, by intersecting Sets 3, 4, and 5, we identified 12 treatment-specific candidate proteins (Figure 3B) whose expression patterns were compatible with IPC-related modulation. Although exploratory, the expression profiles of these proteins showed patterns compatible with IPC-associated modulation. They spanned several biologically relevant pathways, including antioxidant defense (superoxide dismutase 2, mitochondrial (SOD2)), vascular homeostasis regulation (angiopoietin 1 (ANGPT1), heparin-binding EGF-like growth factor (HB-EGF)), inflammation modulation (CD40 ligand (CD40-L), NF-κB essential modulator (NEMO)), and metabolic reprogramming (2,4-dienoyl-CoA reductase 1 (DECR1), hydroxyacid oxidase 1 (HAOX1)).

### Clinical relevance through correlation analysis

Correlation analyses between vascular protein levels and both Essen stroke risk score (ESSEN/ESRS) and NIHSS scores across paired treatment groups (P1). Treatment group 1 (T1) comprised vascular protein measurements taken before intervention with corresponding NIHSS scores, while treatment group 2 (T2) consisted of post-treatment protein levels and associated NIHSS scores. The NIHSS score, a measure that quantifies the severity of neurological deficits, was administered by certified neurologists at baseline (T1) and post-intervention (T2). Similarly, the ESSEN score, which predicts the recurrence risk of ischemic stroke, was calculated based on patients’ medical history and risk factor profiles. By examining correlations between these clinical metrics and vascular proteins, we aimed to identify potential protein biomarkers associated with neurological dysfunction and stroke recurrence. The results revealed six proteins significantly correlated with ESRS, indicating involvement in stroke recurrence risk (Figure 3B). In contrast, thirteen proteins were significantly correlated with NIHSS scores, suggesting relevance to the pathophysiology of neurological impairment (Figure 3B). Notably, higher ESRS-associated protein levels corresponded to increased recurrence risk, while higher NIHSS-associated protein levels linked to lower NIHSS scores, reflecting milder neurological deficits.

Four proteins—macrophage receptor with collagenous structure (MARCO), CD40L, spondin 2, extracellular matrix protein (SPON2), and SOD2—overlapped between NIHSS-correlated proteins and the 12 treatment-specific proteins, supporting their candidacy as potential mediators or markers of treatment response. To evaluate the clinical relevance of these candidates, we constructed a random forest model using all 45 DEPs from the P1 group (Figures 3C and 3D). Variable importance was assessed using mean decrease in accuracy and mean decrease in Gini index. Among these, SOD2 emerged as the top-ranking feature, showing strong negative correlation with NIHSS scores and significantly elevated expression post-treatment. SOD2’s consistent prominence across correlation and classification analyses highlights it as a particularly interesting candidate marker of IPC-related biological effects and a potential target for future mechanistic studies.

### Functional enrichment analysis reveals key molecular networks

**Figure 4:**
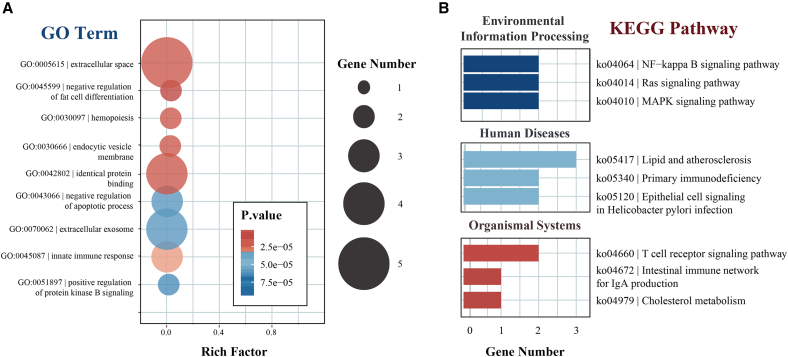
GO and KEGG enrichment analyses of the 12 treatment-specific DEPs (A) Bubble plot showing the top enriched GO terms. These proteins were significantly associated with biological processes including “extracellular space,” “negative regulation of fat cell differentiation,” and “innate immune response.” The size of each bubble indicates the number of genes involved, and color intensity represents statistical significance (*p*-value). (B) KEGG pathway enrichment analysis revealed that the DEPs were involved in multiple functional modules. Key pathways included the Ras signaling pathway and NF-κB signaling pathway (environmental information processing), lipid and atherosclerosis (human diseases), and T cell receptor signaling (organismal systems). The number of genes involved in each pathway is represented by bar length.

GO and KEGG enrichment analyses of the 12 treatment-specific proteins revealed significant enrichment in 305 GO terms and 16 KEGG pathways (Table S1, available at https://doi.org/10.5281/zenodo.16551369). These proteins predominantly localized to the extracellular space and participated in biological processes, including the negative regulation of fat cell differentiation (Figure 4A).

KEGG pathway analysis illuminated critical functional modules: immune regulation through CD40-L and NEMO participation in the primary immunodeficiency pathway (T/B cell activation); metabolic regulation via sortilin 1 (SORT1) and 2,4-dienoyl-CoA reductase 1 (DECR1) contributions to lipid and atherosclerosis pathway (reverse cholesterol transport); and pathology-associated functions through hepatocyte binding protein-epidermal growth factor (HB-EGF) mediated vascular endothelial repair (Figure 4B).

Notably, the Ras signaling pathway (hsa04014) demonstrated the most significant enrichment, involving five DEPs, including ANGPT1 and CD40-L. These proteins also showed significant involvement in NF-κB and T cell receptor signaling pathway (Figure 4B), suggesting central regulatory roles in disease pathogenesis and progression.

**Figure 5:**
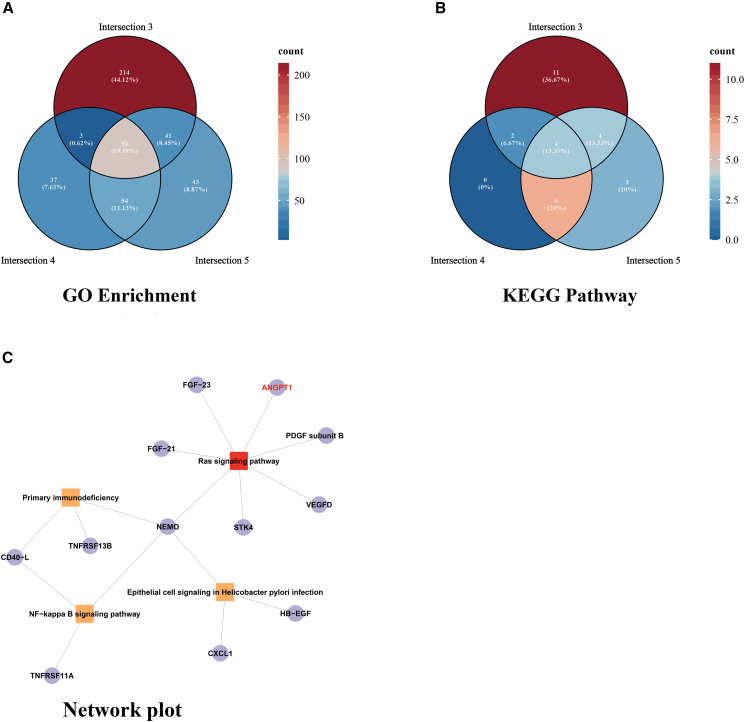
Intersectional enrichment analysis of GO terms and KEGG pathways across three protein sets (A) Venn diagram of significantly enriched GO terms derived from three protein subsets (intersections 3, 4, and 5). A total of 93 GO terms were identified, with 10 terms overlapping across all three sets, representing core biological processes. (B) Venn diagram of significantly enriched KEGG pathways across the same intersections. Four KEGG pathways were identified, among which was the Ras signaling pathway. (C) Consistent enrichment across all sets highlights its central regulatory role. Color gradients represent the number of enriched terms per set, with darker red indicating higher counts.

Intersectional functional enrichment analyses using protein sets 3, 4, and 5 as background (Figures 5A and 5B; Table S2, available at https://doi.org/10.5281/zenodo.16551369) identified 93 significantly enriched GO terms and 4 KEGG pathways. Remarkably, the Ras signaling pathway exhibited the highest significance across all intersection analyses and was consistently enriched in functional analyses of the 12 highly differential proteins (Figure 5C), with ANGPT1 functioning as a crucial regulator in both the Ras signaling pathway and multiple enriched GO terms. Color gradients in Figure 5C represent the number of enriched terms per set, with darker red indicating higher counts. ANGPT1’s central role across multiple analytical approaches strongly suggests its pivotal involvement in both LAA stroke pathogenesis and IPC treatment response.

### Sex-associated differences

In exploratory analyses, most proteins did not differ by sex after FDR correction at either baseline or end of treatment. LOX-1 showed a significant baseline difference between male and female participants after FDR adjustment, whereas no proteins remained significant after FDR correction at the end of treatment (Tables S1 and S2).

## Discussion

Our investigation provides initial insights into the molecular changes associated with IPC in patients with LAA stroke. The proteomics approach employed in this study revealed significant alterations in the expression profiles of cardiovascular-related proteins following intervention, with most proteins showing upregulation in the treatment group compared to controls. Taken together, these exploratory findings indicate that IPC is associated with the modulation of specific protein networks involved in vascular function and neuroinflammation, which may represent candidate pathways through which IPC could influence neurological recovery and which warrant investigation in larger, mechanistic studies.

The identification of 12 treatment-specific proteins with high therapeutic specificity reveals the multifaceted nature of IPC’s biological effects, spanning critical functional domains essential for stroke recovery, including antioxidant defense mechanisms through SOD2, vascular homeostasis regulation via ANGPT1 and HB-EGF, and inflammation modulation through CD40L and NEMO. This diverse protein response is consistent with a coordinated modulation of several pathophysiological pathways, raising the hypothesis that IPC may influence multiple aspects of stroke biology rather than acting through a single mechanism. These exploratory observations require confirmation and mechanistic dissection in larger studies. Notably, these findings align with emerging evidence that effective stroke interventions must address the intricate interplay between vascular repair, inflammation resolution, and neuroplasticity enhancement.

Among these proteins, SOD2 emerged as particularly noteworthy, demonstrating both significant upregulation following treatment and strong negative correlation with NIHSS scores, indicating its potential role in neurological improvement. The prominence of this mitochondrial antioxidant enzyme was unexpected, as mitochondrial antioxidant systems are not typically considered primary targets of external mechanical interventions. This finding aligns with extensive research demonstrating oxidative stress as a central pathophysiological mechanism in stroke,^20^ where excessive reactive oxygen species production contributes to neuronal death, blood-brain barrier disruption, and inflammatory responses. The ability of IPC to enhance endogenous antioxidant capacity through SOD2 upregulation suggests a novel therapeutic mechanism that could complement existing neuroprotective strategies. However, the precise molecular pathways linking external mechanical compression to mitochondrial antioxidant enzyme upregulation remain unclear and represent a critical area for future mechanistic investigation.

Perhaps most remarkably, our functional enrichment analyses consistently identified the Ras signaling pathway as the most significantly enriched pathway across multiple analytical approaches, a discovery that fundamentally contradicts initial expectations that IPC would primarily modulate hemodynamic-related proteins. Ras signaling orchestrates essential cellular processes, including survival, proliferation, differentiation, and angiogenesis, all of which are fundamental to post-stroke neuroplasticity and recovery.^21^ The prominence of this pathway suggests that IPC may be associated with the modulation of Ras-mediated cellular programs involved in tissue repair and functional reorganization. Within this pathway, ANGPT1 emerged as a central regulatory hub, showing significant upregulation following treatment and involvement in numerous enriched biological processes. ANGPT1’s well-established roles in vascular stabilization, endothelial protection, and angiogenesis make it a biologically plausible mediator of IPC-related molecular effects, although this requires direct experimental validation. This finding is consistent with prior studies demonstrating ANGPT1’s neuroprotective effects in experimental stroke models^22^ and suggests that the enhancement of ANGPT1 signaling represents a critical mechanism by which IPC promotes neurovascular repair and recovery.

A particularly informative aspect of our study is the observation of correlations between protein expression changes and clinical metrics. In this pilot cohort, 13 proteins showed nominally significant correlations with NIHSS scores, suggesting they may reflect biological processes linked to neurological status after IPC. The negative correlations—where higher protein levels were associated with lower NIHSS scores—are consistent with a potential relationship between these protein pathways and less severe neurological deficits. However, these findings are exploratory and hypothesis-generating; we did not formally assess the incremental predictive value of these proteins beyond clinical scores, and their utility as biomarkers for monitoring treatment response or predicting outcomes will need to be evaluated in larger, prospectively designed studies.

The overlap of four proteins (MARCO, CD40-L, SPON2, and SOD2) between NIHSS-correlated proteins and our 12 high-differential treatment-specific proteins further supports their potential clinical relevance. This convergence of independent analytical approaches strengthens the evidence for these proteins as key mediators of therapeutic response. In particular, the macrophage receptor MARCO and immune regulator CD40-L suggest immunomodulatory effects of the intervention, consistent with the growing recognition of immune system contributions to stroke recovery.^23^^,^^24^ Recent investigations have further emphasized the role of these immune modulators in neurovascular repair processes following ischemic injury,^25^ aligning with our observations regarding IPC-induced protein expression changes.

From a translational perspective, our findings suggest potential avenues for future clinical application. The identification of treatment-responsive protein candidates provides a preliminary basis for exploring biomarker-guided or personalized approaches to stroke rehabilitation in subsequent studies. This precision medicine approach would optimize resource allocation by identifying likely responders before treatment initiation. Moreover, our characterization of IPC-modulated molecular networks reveals novel targets for combination therapies. Compounds enhancing SOD2 activity or augmenting ANGPT1/Ras pathway signaling could synergize with IPC to amplify therapeutic benefits, representing a promising strategy for maximizing neurological recovery through multi-target approaches.

Our triple orthogonal screening approach represents a methodological innovation for distinguishing true treatment effects from confounding variables in proteomics studies. This framework effectively separated treatment-specific responses from baseline heterogeneity and disease progression, ensuring identified biomarkers reflect intervention-induced changes. The consistency across multiple independent analyses—correlation, functional enrichment, and machine learning—strengthens confidence in the biological relevance of identified proteins and pathways. This rigor is particularly important in stroke research, where patient heterogeneity and complex pathophysiology confound biomarker discovery.

Future research directions should include validation studies in larger cohorts, exploration of longer-term effects, investigation of protein changes in additional tissue types (such as cerebrospinal fluid), and experimental studies to establish causal relationships between the identified proteins and functional recovery. The integration of multi-omics data, including genomics and metabolomics, would provide a more comprehensive understanding of the molecular mechanisms underlying IPC.

The clinical implications of our findings extend beyond biomarker discovery to encompass treatment optimization and patient stratification strategies. The demonstration that IPC induces measurable molecular changes challenges current clinical thinking and may influence treatment protocols, patient selection criteria, and outcome monitoring approaches. Our results suggest that IPC should be considered not merely as a supportive care measure but as an active therapeutic intervention with specific molecular targets. This paradigm shift could lead to more sophisticated treatment algorithms that incorporate biomarker-guided therapy selection and monitoring, ultimately improving patient outcomes through precision medicine approaches.

In conclusion, this pilot randomized study suggests that IPC in patients with LAA stroke is associated with reproducible changes in cardiovascular-related protein networks. We identified a small set of candidate treatment-associated proteins and pathways—including Ras-ANGPT1-related signaling and SOD2-linked antioxidant responses—that may be relevant to vascular repair and neurological recovery. These findings are exploratory and hypothesis-generating; they require validation in larger, independently powered cohorts and dedicated mechanistic studies. Nonetheless, the molecular patterns described here provide a useful starting point for refining IPC protocols, designing mechanistic experiments, and ultimately evaluating whether proteomic signatures can contribute to more personalized approaches to stroke rehabilitation.

### Limitations of the study

Several limitations of our study warrant consideration. First, while our sample size (*n* = 44) enabled the detection of significant protein changes, larger cohorts are essential for validating findings and establishing generalizability across diverse patient populations. Second, this was a pilot study with a relatively small sample size and no formal *a priori* power calculation for the proteomic endpoints. In the context of 92 proteins and multiple related comparisons, our analyses are underpowered to detect small or moderate effects and susceptible to both false-negative and false-positive findings. For this reason, the identified protein signatures and their associations with neurological status should be regarded as exploratory and require confirmation in larger, independently powered cohorts. In addition, sex-associated analyses were exploratory and underpowered. Although LOX-1 differed by sex at baseline after FDR correction, we did not observe robust sex-associated proteomic differences at the end of treatment once controlling for multiple testing; therefore, larger cohorts are needed to clarify whether sex modifies IPC-related proteomic responses. Third, our approach was limited to 92 pre-selected cardiovascular-related proteins. Unbiased proteomics approaches might reveal additional relevant biomarkers not included in the current panel. These limitations may affect the generalizability of the results but do not undermine the core finding that IPC induces specific molecular changes correlating with clinical outcomes. Fourth, because we did not apply a formal panel-wide multiple-testing correction (e.g., FDR) across all 92 proteins, residual false-positive findings cannot be ruled out. The triple orthogonal screening strategy reduces reliance on isolated single-comparison *p*-values but does not replace formal multiple-testing control; accordingly, the protein signatures identified in this study should be regarded as hypothesis-generating and will require confirmation in larger, independent cohorts. Moreover, the study was not powered to detect between-group differences in functional or neurological outcomes, and our analyses of NIHSS change should be regarded as exploratory. Larger, adequately powered trials are needed to determine whether the observed proteomic signatures translate into clinically meaningful benefits.

## Resource availability

### Lead contact

Requests for further information and resources should be directed to and will be fulfilled by the lead contact, Zhi-Xin Huang (hzxd6@163.com).

### Materials availability

This study did not generate new unique reagents.

### Data and code availability

• The data supporting the findings of this study have been deposited in the Zenodo repository and are publicly available at https://doi.org/10.5281/zenodo.16551369.
• This article does not report original code.
• Any additional information required to reanalyze the data reported in this article is available from the lead contact upon request.

## Acknowledgments

The authors thank all study participants and their families. We acknowledge the clinical staff at Guangdong Second Provincial General Hospital for their assistance in patient recruitment and data collection. We further thank Yiyi Huang (Class of 2025, Department of Clinical Medicine, Shandong Second Medical University) for assistance with data organization and figure revisions during article revision and preparation of the responses to reviewers.

This study was supported by Research Funds of Centre for Leading Medicine and Advanced Technologies of IHM (Grant No. 2023IHM01052), Guangzhou Municipal Science and Technology Bureau Key Research and Development Program (2024B03J0436), and the Science Foundation of Guangdong Second Provincial General Hospital (TJGC-2026002). The funders had no role in study design, data collection, analysis, interpretation, or article preparation.

## Author contributions

Z.X.H.: conceptualization, methodology, supervision, project administration, funding acquisition, and writing- original draft preparation. S.L.: conceptualization, investigation, data curation, and writing - original draft preparation. Z.W.: investigation, data curation, and writing - original draft preparation. D.O.: investigation and resources. T.H.: formal analysis, software, and visualization. K.H.: investigation and resources. X.L.: methodology, resources, software, formal analysis, validation, and visualization. All authors contributed to the article and approved the submitted version.

## Declaration of interests

The authors declare no competing interests.

## STAR★Methods

### Key resources table

**Table:** 

REAGENT or RESOURCE	SOURCE	IDENTIFIER
**Deposited data**		

Original Data	the Zenodo repository	https://doi.org/10.5281/zenodo.16551369

**Software and algorithms**		

IBM SPSS Statistics software 27.0	SPSS software	https://www.ibm.com/products/spss-statistics
R studio version 4.4.2	R software	https://cran.rstudio.com/

### Experimental model and study participant details

#### Ethics approval

This study was approved by the Ethics Committee of the Second Hospital of Guangdong Province (approval NO. 2022-KY-KZ-239-03).

#### Study design

This study was a single-blind, randomized controlled trial conducted according to the principles outlined in the Declaration of Helsinki. In this single-blind design, outcome assessors and laboratory personnel who performed protein quantification were blinded to treatment allocation, while therapists administering the pneumatic compression intervention were necessarily aware of group assignment due to the nature of the intervention. Data analysts were also blinded to group allocation until the primary statistical analyses were completed. The study was carried out from April 2023 to June 2024 at a tertiary neurology center. All participants provided informed consent. A total of 44 patients with LAA stroke were ultimately enrolled and allocated using randomization codes generated by specialized statistical software, with 22 patients in the treatment group and 22 patients in the control group. All enrolled participants (*n*=44) were of Han Chinese ethnicity. We noted that the control group included a higher proportion of male patients, which could potentially influence baseline protein expression profiles. To address this, we conducted additional exploratory sex-stratified analyses of protein expression. Specifically, we compared baseline NPX levels between male and female patients, and repeated these comparisons in post-intervention samples. Neurological deficit was assessed using the National Institutes of Health Stroke Scale (NIHSS; range 0–42, higher scores indicating more severe impairment), administered by neurologists who had received specific training and had experience with NIHSS scoring. Vascular risk burden and recurrent vascular risk were summarized using the Essen Stroke Risk Score (ESRS; range 0–9, higher scores indicating greater risk), calculated from each patient’s documented vascular risk factors and medical history.

#### Study population

Eligible patients met the following inclusion criteria: aged ≥18 years, diagnosis of ischemic stroke confirmed clinically and via CTA, DSA, and/or MRA within 72 hours from symptom onset, fulfilling established diagnostic criteria for LAA stroke, and a pre-stroke modified Rankin Scale (mRS) score of 0-1. Exclusion criteria included suspected or confirmed venous thromboembolism (VTE), congestive heart failure, thrombophlebitis in the limb receiving compression, arterial ischemic disease, skin abnormalities (such as ulcers, dermatitis, recent skin grafts, open injuries, or drain placement), severe limb deformity or defects preventing compression sleeve application, and severe allergic reactions to compression sleeves. Patients were withdrawn from the trial if they insisted on withdrawal, experienced serious adverse events, developed hemorrhagic transformation, severe limb infection, or deep vein thrombosis.

### Method details

#### Randomization

The patient randomization process adhered to stringent protocols, with randomization codes sequentially allocated to eligible participants via an automated centralized randomization system. Participants were randomized in a 1:1 ratio to receive either whole-body pneumatic compression therapy twice daily (treatment group) or affected-limb pneumatic compression therapy once daily (control group). The treatment group underwent bilateral pneumatic compression therapy for all four limbs twice daily, with each session lasting 30 minutes over a course of 7 (SD=2) days. In contrast, the control group received unilateral pneumatic compression therapy only on the affected limb once daily for 30 minutes over the same treatment duration. Peripheral venous blood samples (2 mL) were collected in EDTA tubes by a physician at baseline (pre-treatment) and at the conclusion of the pneumatic compression intervention (7 (SD=2) days post-treatment). These samples were centrifuged at 5000 rpm for 5 minutes to extract plasma, which was subsequently stored at -20°C until analysis. Protein quantification was performed by specialized personnel, followed by comprehensive statistical analysis.

#### Pneumatic compression methodology

IPC employs an intermittent pneumatic compression device (IPCD) that cyclically inflates and deflates air chambers, applying rhythmic pressure to the enclosed limbs. This mechanical action induces passive muscle contraction in the compressed limbs, thereby enhancing venous return. In our protocol, the IPCD was calibrated to maintain proximal limb pressure at 80 mmHg, utilizing a graduated compression technique where distal pressure exceeded proximal pressure. The compression cycle consisted of inflation to 120 mmHg maintained for one minute, followed by a 30-second deflation interval, constituting one complete cycle. Each treatment session comprised 15 consecutive cycles. While the treatment group received this therapy twice daily for 30 minutes over 7 (SD=2) days, the control group underwent identical treatment parameters but only once daily. Throughout the intervention, vital signs were meticulously monitored and documented.

#### Analysis of cardiovascular-related biomarkers

Protein quantification was performed using the Olink® Cardiovascular II panel (Olink Proteomics AB, Uppsala, Sweden) following the manufacturer's protocol.^26^ The Proximity Extension Assay (PEA) technology underpinning the Olink platform has been extensively validated^27^ and enables simultaneous analysis of 92 analytes from just 1 μL of sample.

In this methodology, oligonucleotide-labeled antibody probes bind to target proteins, and when probes are in close proximity, the oligonucleotides hybridize. Subsequently, DNA polymerase initiates a proximity-dependent polymerization reaction, generating a unique PCR target sequence. This sequence is then detected and relatively quantified via real-time quantitative PCR (Signature Q100, LC-Bio Technology CO., Ltd., Hangzhou, China). The resulting Ct data underwent rigorous quality control and normalization through multiple internal controls and external quality control samples. The final assay results are expressed as Normalized Protein EXpression (NPX) values, an arbitrary unit on a log2-scale where higher values correspond to increased protein expression levels.

#### Bioinformatics analysis

In our Olink data processing pipeline, we employed the limma package to identify DEPs, implementing a significance threshold of *P*<0.05 to isolate intervention-associated protein signatures. For visual representation of the analytical outcomes, we generated heatmaps and volcano plots using the ggplot2 package in R. Additionally, we conducted Gene Ontology (GO) and Kyoto Encyclopedia of Genes and Genomes (KEGG) enrichment analyses using ggplot2. Specifically, all significantly DEPs were mapped to their corresponding terms or pathways within the GO or KEGG databases, and hypergeometric testing was applied to determine the statistical significance of protein enrichment within specific GO terms or KEGG pathways. We also performed comparative analyses of protein enrichment profiles between different experimental groups, identifying shared differentially enriched functional categories.

To elucidate the relationship between clinical outcomes and intervention efficacy, we conducted Spearman correlation analyses examining the associations between NIHSS scores and DEPs across both treatment groups, thereby identifying proteins strongly correlated with disease progression. Furthermore, group-based differential protein expression analysis was performed using the randomForest package (version 4.7-1.1) in R.

#### Study design and analytical framework

This study conducted a systematic analysis of longitudinal proteomics data from 44 subjects (treatment and control groups) using the Olink Proteomics platform. The research design incorporated two observation timepoints (baseline and post-intervention), with a total of 88 serum samples collected and categorized into four experimental groups: treatment group baseline (T1), treatment group post-intervention (T2), control group baseline (C1), and control group post-intervention (C2). We established a multidimensional research framework integrating both unpaired inter-group comparisons and paired intra-group longitudinal comparisons. This framework encompassed six comparative dimensions: 1) G1: T2 vs. C1; 2) G2: C2 vs. T1; 3) G3: T1 vs. C1; 4) G4: T2 vs. C2; 5) P1: T2 vs. T1; 6) P2: C2 vs. C1. Across these six dimensions, we comprehensively analyzed the expression profiles of 92 cardiovascular-related protein biomarkers (available at https://doi.org/10.5281/zenodo.16551369), with a focus on treatment-specific protein alterations and their biological relevance. Among these comparisons, G3 (T1 vs C1) is used to descriptively characterize baseline proteomic differences between groups, whereas G4 (T2 vs C2), P1 (T2 vs T1), and P2 (C2 vs C1). This framework enabled in-depth exploration of the molecular effects of the intervention and contributed to identifying key mediators underlying its therapeutic mechanism.

Given the exploratory nature of this pilot trial and the targeted panel of 92 pre-specified proteins, we did not apply a formal panel-wide multiple-testing correction such as FDR or Bonferroni. Instead, we reduced the risk of spurious findings by requiring proteins to meet predefined criteria across three orthogonal comparisons (triple screening). Pathway enrichment analyses were used to place the protein-level findings in a broader biological context and to focus interpretation on coherent pathway-level patterns rather than isolated single-protein *p*-values.

### Quantification and statistical analysis

The normality of all continuous variables was assessed using the Shapiro-Wilk test. Normally distributed continuous data are presented as mean and standard deviation (SD), and analyzed using paired-sample t-tests within groups and independent-sample t-tests between groups. Continuous variables that did not follow a normal distribution are reported as median (interquartile range, IQR) and analyzed using nonparametric rank-sum tests (Wilcoxon or Mann–Whitney tests) for both intra-group and inter-group comparisons. Categorical variables are expressed as frequencies (percentages) and were analyzed using the Chi-square test.

Estimated differences between groups are reported with 95% confidence intervals (CIs) and corresponding two-sided *P*-values. A *P*-value ≤0.05 was considered statistically significant, with significance levels marked as ∗ (*P* < 0.05), ∗∗ (*P* < 0.01), and ∗∗∗ (*P* < 0.001). Statistical analyses were performed using IBM SPSS Statistics software (version 27; IBM Corp, Armonk, NY, USA) and R software (version 4.4.2; R Foundation for Statistical Computing, Vienna, Austria).

### Additional resources

The trial was registered at https://www.chictr.org.cn/index.html (registration no. ChiCTR2400088964, principal investigator: Zhi-Xin Huang, date of registration: August 29, 2024).
