# Supplementary material for: Proteomic signatures of intermittent pneumatic compression in patients with large artery atherosclerotic stroke
Source: iScience. 2026 Feb 10;29(3):114915. doi: 10.1016/j.isci.2026.114915 (PMC12936827; doi:10.1016/j.isci.2026.114915)
Supplement: Document S1. Tables S1 and S2 [file mmc1.pdf]

iScience, Volume *29*

Supplemental information

Proteomic signatures of intermittent pneumatic compression in patients with large artery atherosclerotic stroke

Shasha Lei, Zhenzhen Wang, Dandang Ouyang, Tingjing Huang, Kaili Huang, Xinying Li, and Zhi-Xin Huang

Supplementary Table S1. Baseline differential protein expression by sex (male vs female) prior to intermittent pneumatic compression.

Protein	estimate	std.error	statistic	p.value	FDR
BMP.6	0.592	0.463	1.279	0.215	0.645
ANGPT1	0.213	0.268	0.795	0.436	0.759
ADM	-0.026	0.440	-0.060	0.953	0.975
CD40.L	-0.039	0.518	-0.076	0.940	0.975
SLAMF7	-0.297	0.373	-0.795	0.436	0.759
PGF	0.222	0.315	0.706	0.488	0.761
ADAM.TS13	-0.001	0.137	-0.007	0.994	0.994
BOC	0.323	0.254	1.273	0.218	0.645
IL.4RA	0.015	0.198	0.076	0.940	0.975
SRC	0.965	0.469	2.059	0.053	0.573
IL.1ra	-0.161	0.453	-0.356	0.726	0.902
IL6	0.586	0.602	0.974	0.342	0.645
TNFRSF10A	-0.028	0.257	-0.107	0.915	0.975
STK4	0.617	0.380	1.623	0.120	0.628
IDUA	0.412	0.328	1.253	0.225	0.645
TNFRSF11A	0.447	0.332	1.348	0.193	0.645
PAR.1	0.262	0.205	1.277	0.216	0.645
TRAIL.R2	0.318	0.304	1.048	0.307	0.645
PRSS27	0.421	0.267	1.580	0.130	0.628
TIE2	0.195	0.250	0.778	0.446	0.759
TF	0.005	0.266	0.020	0.984	0.994
IL1RL2	-0.074	0.230	-0.321	0.751	0.909
PDGF.subunit.B	0.173	0.086	2.016	0.057	0.573
IL.27	-0.287	0.247	-1.161	0.259	0.645
IL.17D	0.220	0.210	1.045	0.308	0.645
CXCL1	0.502	0.334	1.503	0.148	0.645
LOX.1	1.550	0.350	4.435	0.000	0.023
Gal.9	0.211	0.217	0.976	0.341	0.645
GIF	-0.310	0.592	-0.523	0.607	0.847
SCF	0.309	0.319	0.970	0.344	0.645
IL18	0.310	0.291	1.066	0.299	0.645
FGF.21	-0.250	0.796	-0.314	0.756	0.909
PIgR	-0.016	0.110	-0.149	0.883	0.975
RAGE	-0.229	0.319	-0.717	0.482	0.761
SOD2	-0.061	0.117	-0.522	0.608	0.847
CTRC	-0.997	0.519	-1.921	0.069	0.578
FGF.23	-0.017	0.172	-0.100	0.921	0.975
SPON2	0.062	0.092	0.680	0.505	0.761
GH	-0.443	1.084	-0.408	0.687	0.871
FS	-0.101	0.382	-0.264	0.794	0.913
GLO1	0.446	0.417	1.069	0.298	0.645
CD84	0.277	0.246	1.128	0.273	0.645
PAPPA	0.159	0.271	0.588	0.563	0.810
SERPINA12	-0.904	0.893	-1.013	0.323	0.645
REN	0.967	0.485	1.994	0.060	0.573
DECR1	0.350	0.311	1.124	0.274	0.645
MERTK	0.643	0.234	2.747	0.012	0.286
KIM1	0.988	0.729	1.355	0.191	0.645
THBS2	0.011	0.180	0.058	0.954	0.975

TM	0.416	0.261	1.592	0.127	0.628
VSIG2	0.191	0.449	0.425	0.675	0.871
AMBP	0.199	0.195	1.018	0.321	0.645
PRELP	0.090	0.335	0.267	0.792	0.913
HO.1	-0.172	0.651	-0.265	0.794	0.913
XCL1	-0.507	0.833	-0.609	0.550	0.808
IL16	0.839	0.394	2.131	0.046	0.573
SORT1	0.186	0.177	1.052	0.305	0.645
CEACAM8	1.367	0.382	3.579	0.002	0.086
PTX3	0.493	0.361	1.366	0.187	0.645
PSGL.1	-0.077	0.099	-0.781	0.444	0.759
CCL17	0.495	0.447	1.108	0.281	0.645
CCL3	-0.083	0.409	-0.203	0.841	0.955
MMP7	0.251	0.219	1.144	0.266	0.645
IgG.Fc.receptor.II.b	-0.680	0.483	-1.408	0.174	0.645
ITGB1BP2	0.166	0.244	0.681	0.504	0.761
DCN	0.164	0.230	0.712	0.484	0.761
Dkk.1	0.332	0.185	1.793	0.088	0.579
LPL	-0.312	0.613	-0.508	0.617	0.847
PRSS8	0.381	0.247	1.542	0.139	0.638
AGRP	-0.197	0.438	-0.450	0.658	0.871
HB.EGF	0.804	0.617	1.303	0.207	0.645
GDF.2	0.648	0.381	1.702	0.104	0.600
FABP2	-0.203	0.469	-0.433	0.670	0.871
THPO	0.084	0.272	0.309	0.761	0.909
MARCO	-0.242	0.142	-1.703	0.104	0.600
GT	-0.969	0.481	-2.015	0.058	0.573
BNP	0.145	0.240	0.603	0.553	0.808
MMP12	0.546	0.419	1.306	0.206	0.645
ACE2	0.839	0.289	2.904	0.009	0.269
PD.L2	0.266	0.659	0.403	0.691	0.871
CTSL1	-0.178	0.244	-0.732	0.472	0.761
hOSCAR	-0.019	0.223	-0.086	0.932	0.975
TNFRSF13B	-0.023	0.244	-0.093	0.927	0.975
TGM2	0.756	0.652	1.158	0.260	0.645
LEP	-0.510	0.574	-0.889	0.385	0.708
CA5A	0.924	0.512	1.804	0.086	0.579
HSP.27	0.369	0.492	0.750	0.462	0.761
CD4	-0.105	0.247	-0.427	0.674	0.871
NEMO	0.934	0.502	1.858	0.078	0.579
VEGFD	-0.342	0.331	-1.032	0.315	0.645
PARP.1	0.166	0.166	0.999	0.330	0.645
HAOX1	1.561	0.790	1.975	0.062	0.573

Abbreviations: SE, standard error; FDR, false discovery rate.

Supplementary Table S2. Comparison of male vs female patients after intermittent pneumatic compression (end of treatment).

Protein	estimate	std.error	statistic	p.value	FDR
BMP.6	0.071	0.324	0.219	0.829	0.975
ANGPT1	-0.096	0.114	-0.840	0.411	0.975
ADM	-0.499	0.472	-1.058	0.303	0.975
CD40.L	-0.524	0.352	-1.490	0.152	0.975
SLAMF7	0.026	0.258	0.102	0.920	0.975
PGF	0.052	0.168	0.311	0.759	0.975
ADAM.TS13	-0.120	0.097	-1.240	0.229	0.975
BOC	0.161	0.147	1.098	0.285	0.975
IL.4RA	0.095	0.201	0.472	0.642	0.975
SRC	-0.134	0.280	-0.478	0.638	0.975
IL.1ra	-0.196	0.352	-0.556	0.584	0.975
IL6	-0.083	0.400	-0.207	0.838	0.975
TNFRSF10A	0.027	0.143	0.189	0.852	0.975
STK4	0.024	0.313	0.078	0.939	0.975
IDUA	-0.352	0.268	-1.315	0.203	0.975
TNFRSF11A	-0.114	0.255	-0.448	0.659	0.975
PAR.1	-0.085	0.187	-0.455	0.654	0.975
TRAIL.R2	-0.015	0.194	-0.075	0.941	0.975
PRSS27	-0.509	0.249	-2.041	0.055	0.975
TIE2	0.141	0.262	0.539	0.596	0.975
TF	-0.109	0.106	-1.026	0.317	0.975
IL1RL2	-0.061	0.178	-0.342	0.736	0.975
PDGF.subunit.B	-0.006	0.063	-0.094	0.926	0.975
IL.27	-0.145	0.264	-0.548	0.590	0.975
IL.17D	-0.059	0.119	-0.493	0.628	0.975
CXCL1	-0.258	0.262	-0.983	0.337	0.975
LOX.1	-0.103	0.202	-0.507	0.618	0.975
Gal.9	0.003	0.239	0.011	0.991	0.991
GIF	0.044	0.361	0.122	0.904	0.975
SCF	0.120	0.271	0.444	0.662	0.975
IL18	0.332	0.288	1.153	0.263	0.975
FGF.21	0.137	0.740	0.186	0.854	0.975
PIgR	0.151	0.102	1.487	0.153	0.975
RAGE	0.010	0.222	0.046	0.964	0.975
SOD2	-0.033	0.071	-0.467	0.646	0.975
CTRC	0.017	0.305	0.057	0.955	0.975
FGF.23	-0.100	0.143	-0.698	0.493	0.975
SPON2	0.007	0.084	0.082	0.935	0.975
GH	-0.521	0.703	-0.742	0.467	0.975
FS	-0.079	0.286	-0.278	0.784	0.975
GLO1	0.059	0.327	0.180	0.859	0.975
CD84	-0.232	0.180	-1.289	0.212	0.975
PAPPA	0.153	0.143	1.067	0.299	0.975
SERPINA12	-0.094	0.704	-0.134	0.895	0.975
REN	0.507	0.358	1.416	0.172	0.975
DECR1	-0.650	0.444	-1.464	0.159	0.975
MERTK	0.418	0.240	1.741	0.097	0.975
KIM1	-0.536	0.295	-1.813	0.085	0.975
THBS2	-0.048	0.137	-0.355	0.727	0.975

TM	0.220	0.185	1.186	0.250	0.975
VSIG2	0.157	0.254	0.616	0.545	0.975
AMBP	0.099	0.100	0.993	0.332	0.975
PRELP	0.024	0.390	0.062	0.952	0.975
HO.1	0.276	0.505	0.547	0.591	0.975
XCL1	-0.094	0.571	-0.165	0.871	0.975
IL16	0.201	0.338	0.595	0.559	0.975
SORT1	-0.015	0.158	-0.094	0.926	0.975
CEACAM8	-0.260	0.322	-0.806	0.430	0.975
PTX3	0.170	0.267	0.637	0.531	0.975
PSGL.1	-0.073	0.085	-0.855	0.403	0.975
CCL17	0.042	0.568	0.073	0.942	0.975
CCL3	-0.282	0.321	-0.877	0.391	0.975
MMP7	0.051	0.241	0.213	0.834	0.975
IgG.Fc.receptor.II.b	-0.496	0.191	-2.597	0.017	0.792
ITGB1BP2	-0.065	0.153	-0.426	0.675	0.975
DCN	0.148	0.189	0.785	0.442	0.975
Dkk.1	-0.113	0.141	-0.801	0.433	0.975
LPL	-0.177	0.499	-0.355	0.726	0.975
PRSS8	0.152	0.186	0.818	0.423	0.975
AGRP	-0.135	0.247	-0.548	0.590	0.975
HB.EGF	-0.634	0.355	-1.787	0.089	0.975
GDF.2	-0.053	0.302	-0.177	0.861	0.975
FABP2	0.067	0.388	0.172	0.865	0.975
THPO	-0.119	0.256	-0.465	0.647	0.975
MARCO	-0.074	0.135	-0.547	0.590	0.975
GT	-0.435	0.278	-1.566	0.133	0.975
BNP	-0.018	0.118	-0.155	0.879	0.975
MMP12	0.251	0.313	0.802	0.432	0.975
ACE2	0.362	0.462	0.785	0.442	0.975
PD.L2	0.204	0.618	0.331	0.744	0.975
CTSL1	0.165	0.195	0.846	0.407	0.975
hOSCAR	0.120	0.164	0.730	0.474	0.975
TNFRSF13B	-0.017	0.194	-0.088	0.931	0.975
TGM2	-0.617	0.385	-1.601	0.125	0.975
LEP	-1.911	0.558	-3.427	0.003	0.245
CA5A	-0.959	0.666	-1.439	0.166	0.975
HSP.27	-0.295	0.381	-0.775	0.447	0.975
CD4	0.175	0.207	0.847	0.407	0.975
NEMO	0.051	0.380	0.133	0.895	0.975
VEGFD	0.056	0.211	0.266	0.793	0.975
PARP.1	-0.133	0.148	-0.901	0.378	0.975
HAOX1	0.077	0.996	0.077	0.939	0.975

Abbreviations: SE, standard error; FDR, false discovery rate.
